# 5-aza-2′-deoxycytidine (DAC) treatment downregulates the HPV E6 and E7 oncogene expression and blocks neoplastic growth of HPV-associated cancer cells

**DOI:** 10.18632/oncotarget.10631

**Published:** 2016-07-16

**Authors:** Maximilian Stich, Lennard Ganss, Jens Puschhof, Elena-Sophie Prigge, Miriam Reuschenbach, Ana Guiterrez, Svetlana Vinokurova, Magnus von Knebel Doeberitz

**Affiliations:** ^1^ Department of Applied Tumor Biology, Institute of Pathology, University of Heidelberg, Germany; ^2^ Clinical Cooperation Unit Applied Tumor Biology, German Cancer Research Center (DKFZ), Heidelberg, Germany; ^3^ Institute of Carcinogenesis, NN Blokhin Cancer Research Center, Moscow, Russia

**Keywords:** HPV, DNA demethylation, 5-aza-2′-deoxycytidine (DAC), miR-375, upstream regulatory region (URR)

## Abstract

High-risk human papillomaviruses (hr HPVs) may cause various human cancers and associated premalignant lesions. Transformation of the host cells is triggered by overexpression of the viral oncogenes E6 and E7 that deregulate the cell cycle and induce chromosomal instability. This process is accompanied by hypermethylation of distinct CpG sites resulting in silencing of tumor suppressor genes, inhibition of the viral E2 mediated control of E6 and E7 transcription as well as deregulated expression of host cell microRNAs. Therefore, we hypothesized that treatment with demethylating agents might restore those regulatory mechanisms. Here we show that treatment with 5-aza-2′-deoxycytidine (DAC) strongly decreases the expression of E6 and E7 in a panel of HPV-transformed cervical cancer and head and neck squamous cell carcinoma cell lines. Reduction of E6 and E7 further resulted in increased target protein levels including p53 and p21 reducing the proliferation rates and colony formation abilities of the treated cell lines. Moreover, DAC treatment led to enhanced expression of tumor the suppressive miRNA-375 that targets and degrades E6 and E7 transcripts. Therefore, we suggest that DAC treatment of HPV-associated cancers and respective precursor lesions may constitute a targeted approach to subvert HPV oncogene functions that deserves testing in clinical trials.

## INTRODUCTION

High-risk human papillomavirus (HR-HPV) infections may cause various human cancers in particular of the female anogenital tract but also of the oropharynx and other epithelial sites [1]. HPV-associated cancer cells are addicted to the consistent expression of two HPV-encoded oncogenes referred to as E6 and E7 [2, 3]. Their gene products interfere with a variety of host proteins resulting in the deregulation of the cell cycle and chromosomal instability upon expression in replicating cells [4, 5]. Key oncogenic functions of these proteins are the E6-mediated degradation of the p53 protein driving the abrogation of proapoptotic pathways and the E7-mediated degradation of the retinoblastoma protein (pRB) causing an excess release of E2F transcription factors and activation of cell cycle progression [6–10]. Furthermore, activation of the viral oncogenes induces a substantial overexpression of the cyclin dependent kinase inhibitor p16^INK4a^, which has therefore been extensively used as a biological marker for “transforming HPV-infections” [11]. Despite intense research activities on the biochemistry and molecular biology of the viral oncogene products and their functions in HPV-transformed cells no targeted therapies interfering with the activity of E6 and E7 yet reached the level of broader clinical testing.

Accumulating evidence now suggests that epigenetic factors may play an important role in the activation of the HPV oncogenes [12]. The transcription regulating functions of the E2 protein, the key regulator of HPV early gene expression, appears to be strongly affected by the methylation status of CpG dinucleotides within the E2 binding sites (E2BSs) located in the viral upstream regulatory region (URR) [13–18]. Furthermore, activation of the viral oncogenes and subsequent transformation of the host cells is accompanied by a substantial shift in the methylation pattern of distinct CpG dinucleotides in the viral as well as in the host cell genome [19–23].

Another recently described mechanism that potentially contributes to the activation of E6 and E7 is the downregulation of miR-375 [24–26]. The expression of this microRNA (miRNA) was shown to be repressed by methylation of CpGs located in its promoter region. Enhanced methylation of this region could be observed in transforming HPV-infections including preneoplastic lesions as well as invasive carcinomas [27]. Consequently, expression of miR-375 decreases during HPV-mediated cervical transformation [25, 26]. Interestingly, miR-375 was shown to suppress the expression of multiple host cellular and viral oncogenic factors including the transcription factor SP1, the E6-associated protein (E6AP) and the HPV 16 and 18 oncogenes E6 and E7 [24, 25]. Thereby, miR-375 may contribute to the intracellular surveillance to prevent the oncogenic activity and is therefore suggested to play a tumor suppressive role especially in HPV-associated cancers [28].

Overall, these findings suggest that increasing CpG methylation plays an important role during HPV-mediated transformation. Therefore, we hypothesized that treatment of HPV-transformed cells with the demethylating agent 5-aza-2′-deoxycytidine (DAC) may restore various regulatory mechanisms abrogated by hypermethylation. The cytidine analog DAC is incorporated into the DNA during replication and irreversibly binds to DNA methyltransferase 1 (DNMT1) [29–31]. DAC (decitabine) has been approved for the treatment of myelodysplastic syndrome (MDS) by the U.S. Food and Drug Administration (FDA) in 2006, however, it also shows promising effects in the treatment of some solid cancers [32, 33]. Here we analyzed the effects of DAC treatment on the expression of the HPV oncogenes E6 and E7, as well as their target proteins including p53 and p21 in a panel of six HPV-transformed cell lines including HPV 16 and 18 positive cervical cancer cells as well as HPV 16 positive head and neck squamous cell carcinoma (HNSCC) cell lines. Furthermore, we monitored the neoplastic growth properties of the cells during DAC treatment. Finally, demethylation of E2BS 3 and 4 and reexpression of miR-375 were analyzed to evaluate their roles in regulating E6 and E7 expression.

## RESULTS

### DAC treatment reduces E6 and E7 oncogene expression in HPV 16 and 18 transformed cervical carcinoma and HNSCC cell lines

In order to analyze the effect of demethylating agents on the expression of the HPV oncogenes E6 and E7, we treated cervical carcinoma and HNSCC cell lines for 72 hours with different concentrations of DAC ranging from 0.1 μM to 1.0 μM. Four HPV 16 transformed cell lines were selected: the cervical carcinoma cell lines CaSki and SiHa as well as the HNSCC cell lines UM-SCC-47 and UM-SCC-104. To extend the study to HPV 18 infected cell lines, we additionally included the cervical carcinoma cell lines C4-1 and SW756 (Table 1).

**Table 1:** Summary of HPV type, origin, E2BS methylation level and the status of the E2 gene in the included cell lines

Cell line	HPV Type	Origin	E2BS Methylation	Status E2 Gene
**CaSki**	16	Cervix	High	Intact
**SiHa**	16	Cervix	Low	Disrupted
**UM-SCC-47**	16	Lateral Tongue	High	Disrupted [52], but E2 expression detected [53]
**UM-SCC-104**	16	Floor of Mouth	Low	Disrupted
**C4-1**	18	Cervix	Low	Disrupted
**SW756**	18	Cervix	Low	Disrupted

First, we assessed the demethylating effect of DAC treatment by measuring DNA methylation in the retrotransposon Long Interspersed Nuclear Element 1 (LINE1), which is heavily methylated in most tissues and cell lines and often used as a surrogate marker for global DNA methylation levels [34]. DAC treatment for 72 hours resulted in consistent demethylation of CpGs located in the LINE1 transposable element in all included cell lines (Figure S1).

**Figure 1:**
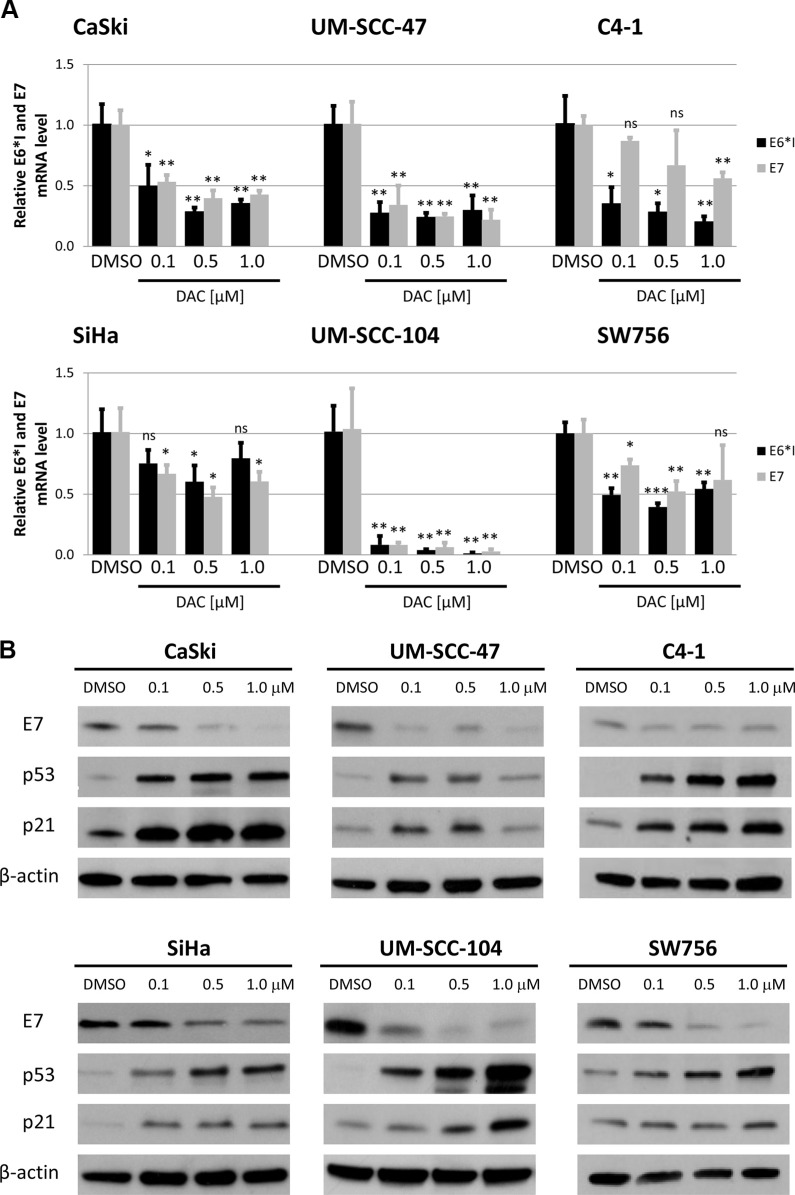
DAC treatment reduces HPV oncogene expression and leads to increased levels of the downstream factors p53 and p21 (**A**) Treatment of CaSki, SiHa, UM-SCC-47, UM-SCC-104, C4-1 and SW756 with DAC for 72 hours resulted in decreased E6*I and E7 mRNA levels as quantified by RT-qPCR. The results are presented as mean values from at least three independent experiments. Error bars represent the according standard deviation and *p* values were calculated by performing Student's *t*-test. DMSO treated cells were used as a reference control. *p < 0.05, **p < 0.01, ***p < 0.001 and ns: not significant. (**B**) Western Blot analysis resulted in reduced E7 protein levels in all tested cell lines after DAC treatment for 72 hours. In addition, levels of p53 and p21 increased after DAC treatment. β-actin was used as an internal loading control.

After confirming the demethylating effect of the treatment we quantified the expression of E6 and E7 oncogenes. RT-qPCR revealed a strong reduction in E6*I and E7 mRNA levels [35] after DAC treatment in CaSki, UM-SCC-47, UM-SCC-104, and SW756 cells (Figure 1A). The strongest effects were observed in CaSki, UM-SCC-47 and UM-SCC-104 cells resulting in HPV oncogene mRNA downregulation of up to 80 fold (UM-SCC-104, E6*I, 1.0 μM DAC). In SiHa and C4-1 the HPV oncogene mRNA levels were only slightly reduced compared to the expression in DMSO treated control cells.

Reduction of HPV oncogene mRNA expression after DAC treatment also resulted in decreased E7 protein levels in CaSki, UM-SCC-47, UM-SCC-104 and SW756 cells (Figure 1B). Although, E6*I and E7 mRNA expression was only moderately reduced in SiHa cells, E7 protein levels were substantially decreased. Similar to the HPV oncogene mRNA expression E7 protein levels were only slightly reduced in C4-1 cells. Additionally, the protein levels of the cell cycle regulators p53 and p21, which are downstream targets of the HPV E6 oncoprotein increased after DAC treatment in all tested cell lines. Based on these results, we conclude that treatment with the demethylating agent DAC results in downregulation of E6 and E7 oncogene expression and reactivation of their repressed target proteins p53 and p21.

### DAC treatment inhibits proliferation and colony formation of HPV-transformed cell lines

**Figure 2:**
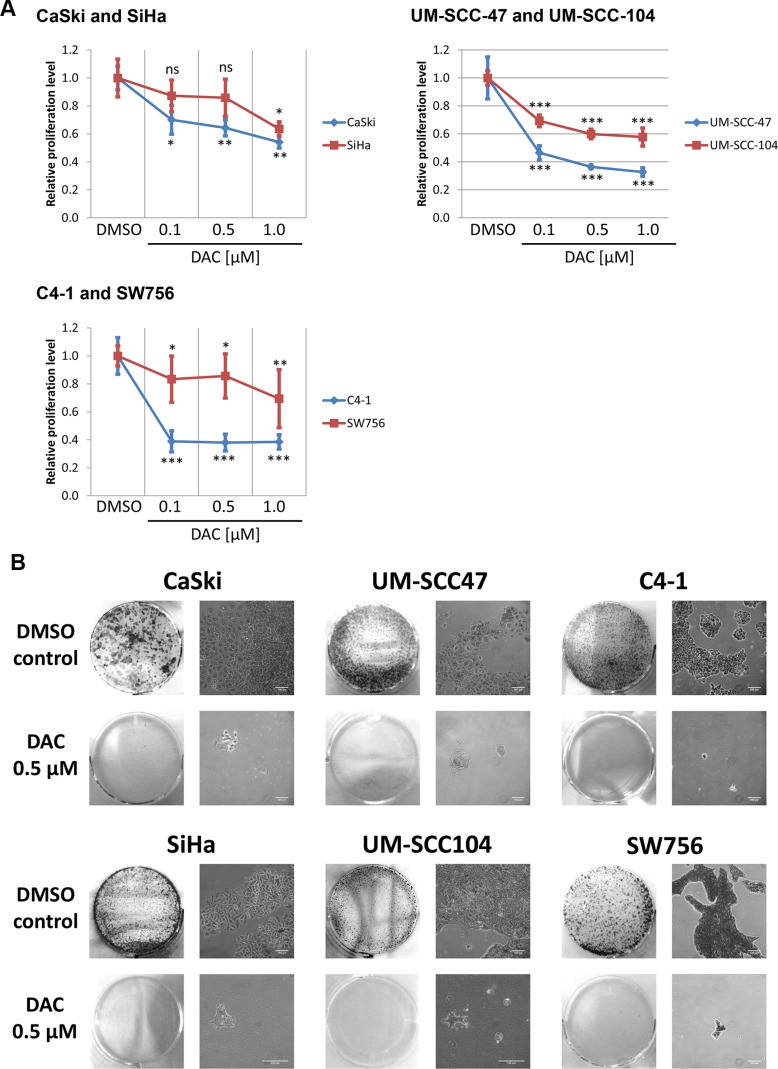
DAC treatment represses proliferation and colony formation of HPV 16 and 18 positive cervical carcinoma and HNSCC cell lines (**A**) Proliferation of cells after 72 hours DAC treatment was determined by measuring the DNA content. The diagram shows the mean proliferation levels for at least three independent experiments and the according standard deviation. Student`s *t*-test was performed to calculate *p* values using DMSO treated cells as a reference control. *p < 0.05, **p < 0.01, ***p < 0.001 and ns: not significant. (**B**) Cells treated for 72 hours with 0.5 μM DAC or with the solvent DMSO were transferred after treatment into 6 well plates and cultured without treatment for 7 days. Afterwards the cells were stained using crystal violet to monitor colony formation. Representative images are shown. The scale bars reflect 100 μm.

Next, we tested whether DAC treatment affects proliferation and colony formation of the included cell lines. As shown in Figure 2A treatment with DAC reduced proliferation of all tested cell lines. Inhibition of proliferation could already be observed after treating the cell lines with 0.1 μM DAC, however, stronger effects were obtained by increasing the DAC concentration to 0.5 and 1.0 μM. Proliferation was most effectively reduced in CaSki, UM-SCC-47, UM-SCC-104 and C4-1 cells, whereas moderate effects were observed for SiHa and SW756 cells. Similarly, colony formation capacities of cells treated with 0.5 μM DAC were impaired in comparison to DMSO treated control cells as shown by crystal violet staining (Figure 2B). Taken together, DAC treatment significantly impairs proliferation and colony formation capacities in HPV-transformed cell lines.

### DAC treatment results in demethylation of E2BS 3 and 4 in CaSki and UM-SCC-47

**Figure 3:**
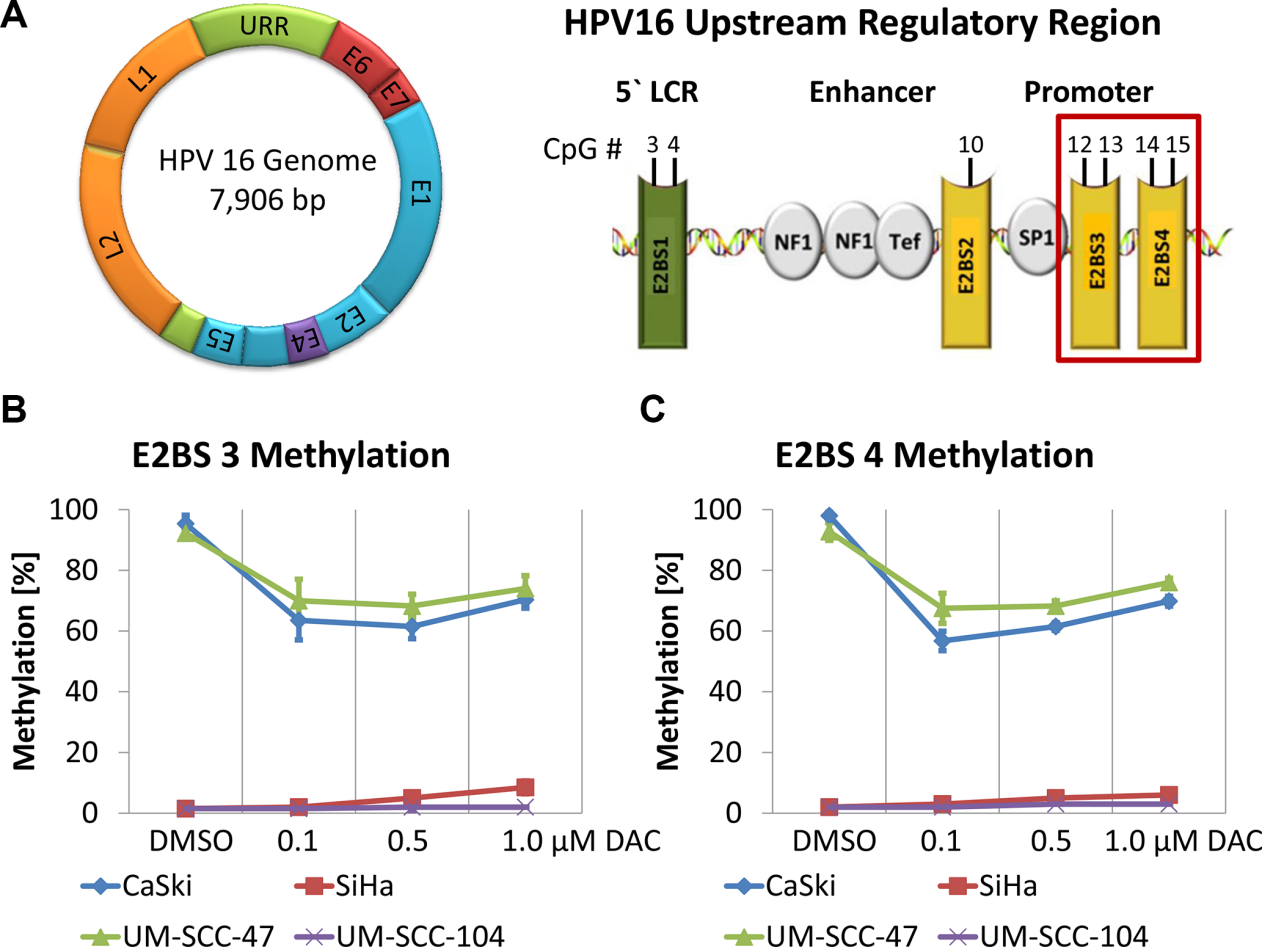
Demethylation of E2BS 3 and 4 in the HPV 16 URR of CaSki and UM-SCC-47 cells after DAC treatment (**A**) Schematic illustration of the HPV 16 genome and the URR highlighting E2BS 3 and 4. CpG methylation levels of E2BS 3 (**B**) and 4 (**C**) in CaSki, SiHa, UM-SCC-47 and UM-SCC-104 after treatment with DAC. The results are presented as mean methylation levels of the two CpG dinucleotides located in E2BS 3 (nucleotide position: 37 and 43) and in E2BS 4 (nucleotide position: 52 and 58) as obtained by pyrosequencing. Error bars represent the standard deviation of the mean methylation level.

Expression of the HPV early genes including E6 and E7 is mainly regulated by the viral E2 protein, which interacts with four E2BSs located in the HPV URR (Figure 3A). Binding of E2 to E2BS 3 and 4, which are located proximal to the TATA box of the E6 and E7 promoter represses oncogene transcription through the displacement of Sp1 and TBP from their binding sites [36, 37]. However, methylation of CpG dinucleotides in the E2BSs was shown to prevent the interaction of E2 resulting in deregulated E6 and E7 expression [13, 17, 18].

To analyze whether DAC treatment might restore this regulatory mechanism we determined the methylation levels of E2BS 3 and 4 in CaSki, SiHa, UM-SCC-47 and UM-SCC-104 cells. E2BS 3 and 4 methylation levels in CaSki and UM-SCC-47 cells were reduced from more than 92% in DMSO treated control cells to 56 - 76% after DAC treatment (Figure 3B and 3C). Low E2BS 3 and 4 methylation levels were detected in SiHa and UM-SCC-104 cells. These levels were not further affected by the treatment.

Moreover, CaSki cells contain intact E2 further substantiating the hypothesis that DAC treatment might restore the E2-mediated repression of E6 and E7 in cells that show high E2BS methylation and express intact E2 as reported by Fernandez and colleagues [38]. However, reduction of E6 and E7 levels could also be observed in the other cell lines, which either do not express intact E2 proteins due to the disruption of the E2 gene during the integration of the viral genome into the host cell DNA or show low E2BS methylation levels (Table 1 and Figure 3B and 3C). Therefore, additional mechanisms seem to be involved in decreasing the E6 and E7 oncogene expression independent of the presence of E2 proteins.

### Expression of tumor suppressive miR-375 is reactivated after DAC treatment and targets E6 and E7 transcripts

Recently, miR-375 was reported to play an important role in the regulation of HPV 16 and 18 oncogene expression as this miRNA was shown to target E6 and E7 transcripts [24]. Expression of miR-375 was found to be repressed by methylation of CpG dinucleotides located in its promoter region [27]. Therefore, we speculated that treatment with DAC might activate the expression of miR-375 in the included cell lines subsequently targeting HPV E6 and E7 transcripts.

**Figure 4:**
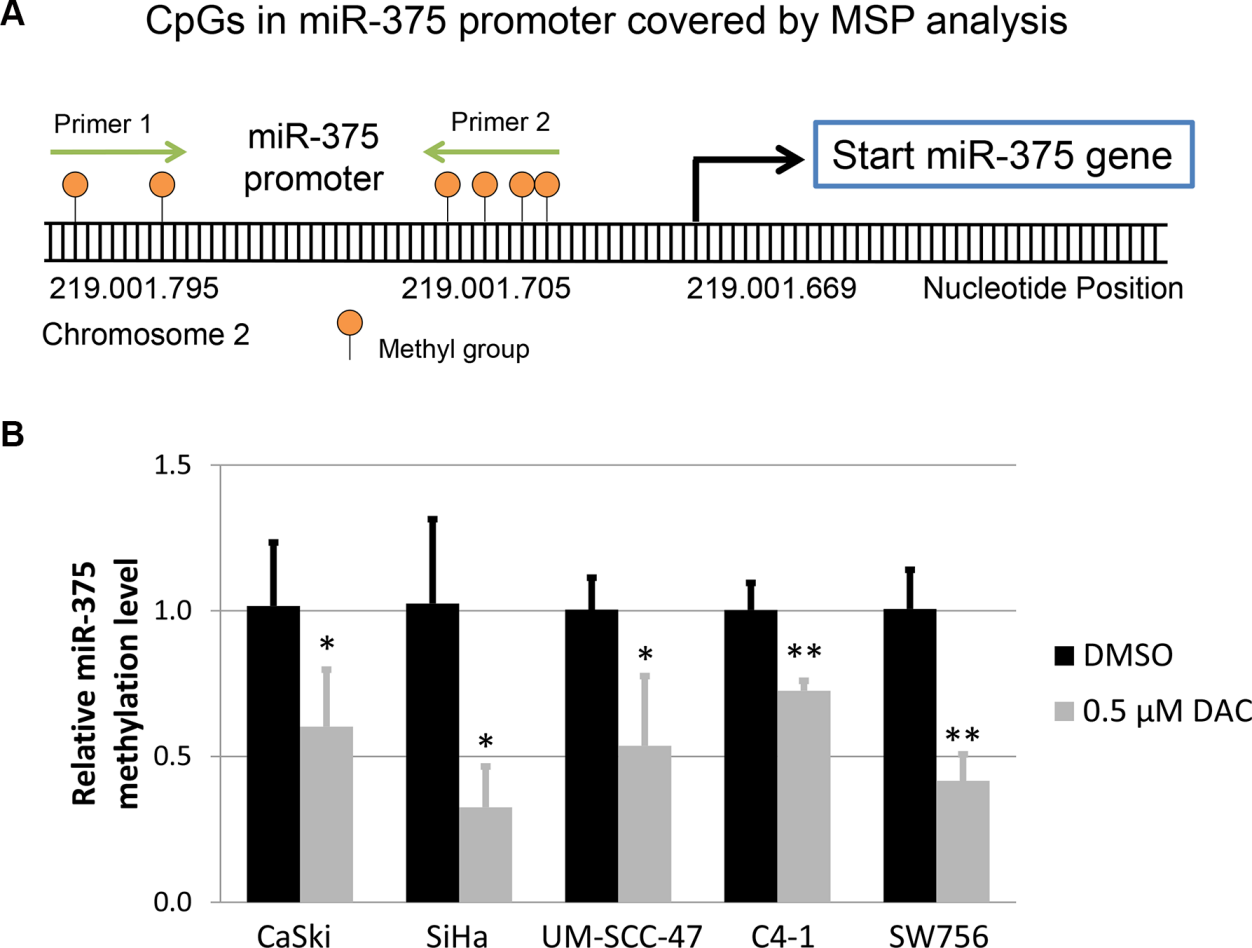
Methylation level of miR-375 promoter under DAC treatment (**A**) Schematic illustration of the methylation-specific qPCR (MSP) assay performed to quantify the methylation level in the miR-375 promoter. The graphic shows six CpG dinucleotides located in the miR-375 promoter, which were covered by the primers used for MSP. These primers were designed to amplify bisulfite converted and methylated template DNA. (**B**) Comparison of the methylation level in the miR-375 promoter region between HPV-transformed cell lines treated with 0.5 μM DAC and DMSO using MSP analysis. The bisulfite and methylation-specific primers were designed to cover CpG dinucleotides located in close proximity to the start of the miR-375 gene (as shown in A). As a reference gene bisulfite converted and unmethylated β-actin sequences were amplified indicating successful bisulfite conversion and sufficient DNA quality. The results are presented as mean methylation levels from at least three independent treatments. The error bars indicate the according standard deviation and Student`s *t*-test was used to calculate *p* values. *p < 0.05 and **p < 0.01.

To test this hypothesis we analyzed the methylation levels of CpG dinucleotides located in the miR-375 promoter region by performing methylation-specific qPCR as described previously [27]. Treatment with 0.5 μM DAC resulted in demethylation of the miR-375 promoter region in CaSki, SiHa, UM-SCC-47, C4-1 and SW756 cells in comparison to DMSO treated control cells (Figure 4).

**Figure 5:**
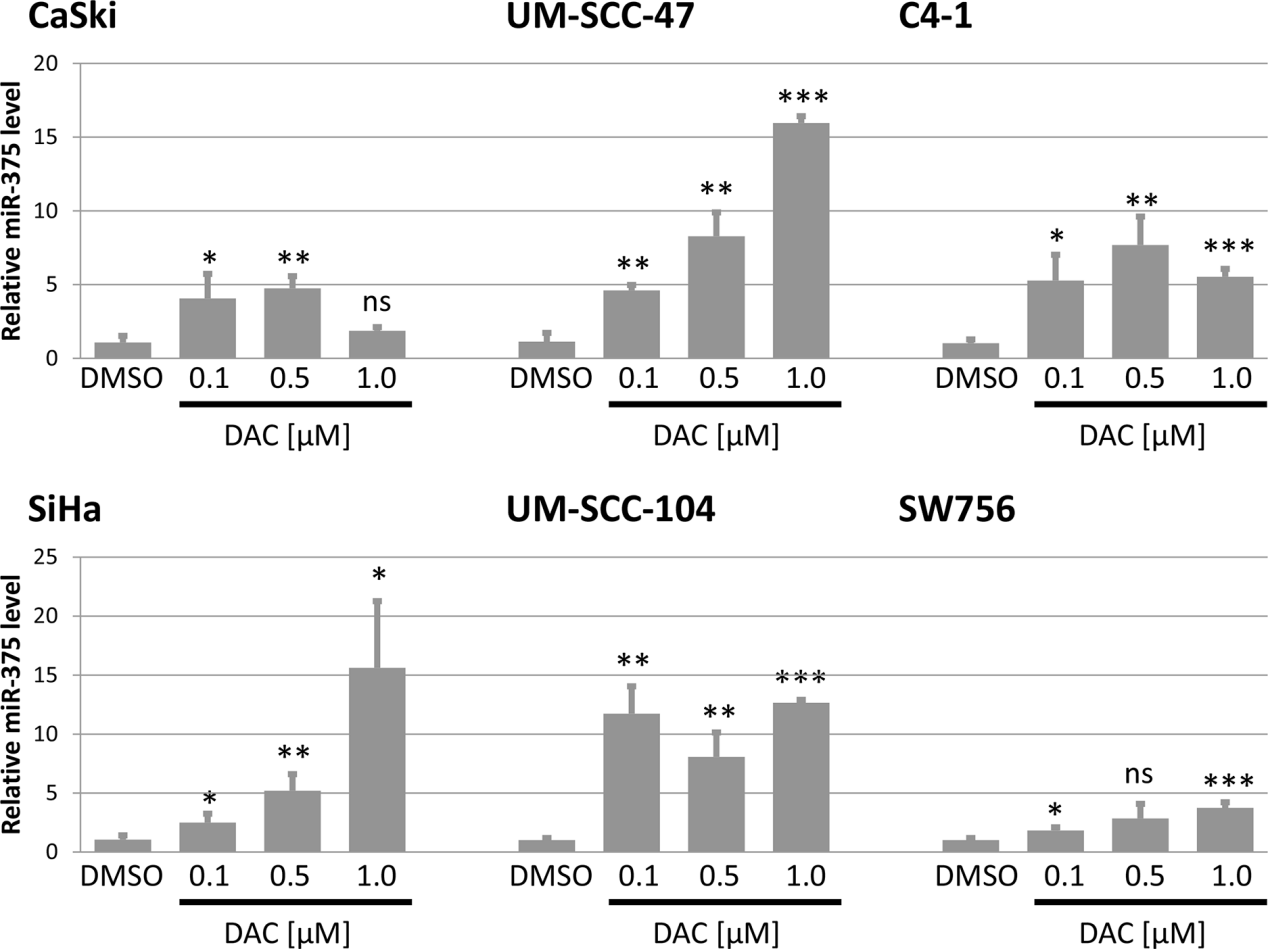
Expression of miR-375 is reactivated after treatment with DAC in all tested cell lines Quantification of miR-375 in DAC treated cell lines was performed using TaqMan qPCR assays. miR-375 levels were calculated relative to the solvent DMSO (mock) and snRNA U6 was used as internal loading control. Data are shown as mean miR-375 levels from at least three independent experiments and the error bars reflect the according standard deviation. Student`s *t*-test was performed to calculate *p* values by using DMSO as a reference control. *p < 0.05, **p < 0.01, ***p < 0.001 and ns: not significant.

In the next step, we analyzed the effect of DAC treatment on the expression of miR-375. In all tested cell lines we detected a significant increase in miR-375 expression after DAC treatment (Figure 5). In CaSki cells treatment with 0.5 μM DAC resulted in an almost 5 fold increase of miR-375 levels compared to DMSO treated control cells. Similar effects could be observed in SiHa and UM-SCC-47 cells as increasing levels of DAC led to continuously elevated miR-375 concentrations (up to 15 fold) compared to cells treated with the solvent DMSO. Treatment of UM-SCC-104 also resulted in a strong increase of miR-375 expression levels. The effects of DAC treatment on miR-375 expression in the HPV 18 transformed cell lines were similar. Treatment of C4-1 cells with 0.5 μM DAC led to a more than 7 fold increase of miR-375 levels in comparison to DMSO and in SW756 cells miR-375 levels increased continuously with elevating DAC concentrations (up to 3.7 fold increase).

**Figure 6:**
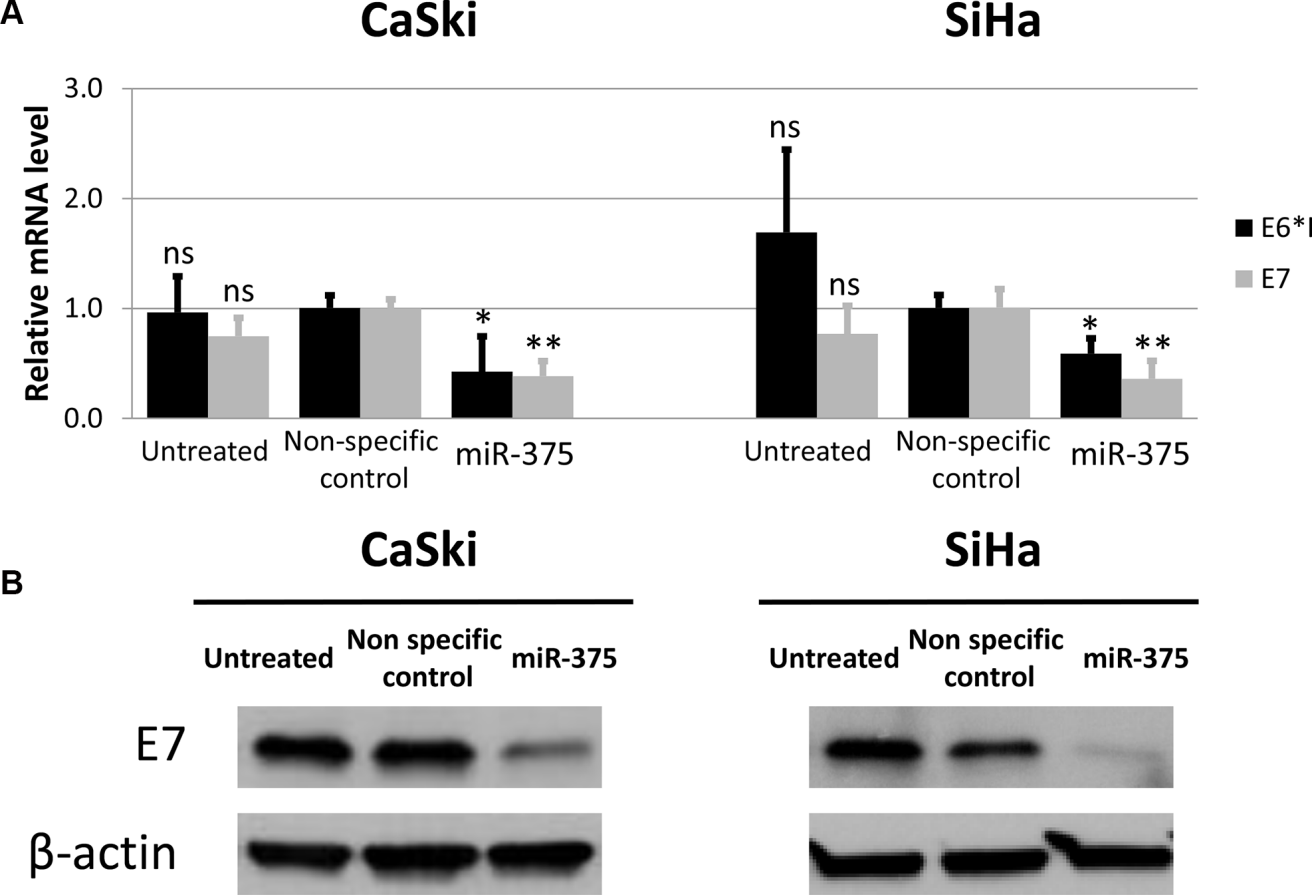
Transfection of miR-375 mimics reduces HPV oncogene levels in CaSki and SiHa cells (**A**) Expression of endogenous E6*I and E7 mRNA in CaSki and SiHa cells transfected with miR-375 mimics was quantified using RT-qPCR. Final concentration of 100 nM was used for miR-375 mimics as well as 25 nM for non-specific control miRNAs. The results are indicated as mean E6*I and E7 mRNA levels measured 48 hours after transfection and the according standard deviation is represented by the error bars. *P* values were calculated by utilizing Student`s *t*-test using non-specific control as a reference. *p < 0.05, **p < 0.01 and ns: not significant. (**B**) E7 protein levels in CaSki and SiHa cells 48 hours after transfection with miR-375. Final concentration of 100 nM was used for miR-375 mimics as well as 25 nM for non-specific control miRNAs. Actin was used as an internal control.

To test the effects of increased miR-375 expression levels on the steady state level of E6 and E7 mRNA transcripts we transfected the HPV 16 transformed cell lines CaSki and SiHa with miR-375. 48 hours after transfection the E6*I and E7 transcript levels were significantly reduced in both cell lines (Figure 6A) and Western blot analysis resulted in reduced levels of the E7 gene product (Figure 6B).

Based on these results we conclude that the reactivation of miR-375 expression in HPV 16 and 18 transformed cervical carcinoma and HNSCC cell lines after treatment with DAC is linked to the downregulation of the E6 and E7 oncogene expression.

## DISCUSSION

Alterations in DNA methylation patterns play an important role in the development and progression of HPV-mediated cancers [21, 23]. Methylation of the viral genome was observed to progressively increase from symptom free HPV carriers to primary carcinomas [38]. Especially methylation of the URR was shown to be altered in high grade precancers and invasive cancers, potentially being involved in the deregulated expression of the HPV oncogenes [16]. Previous studies highlighted the role of the E2 protein in regulating E6 and E7 transcription and showed that elevated methylation, particularly of the proximal E2BS 3 and 4 may abrogate this regulatory mechanism by preventing E2 binding [17, 18]. However, during HPV-induced carcinogenesis changes in the DNA methylation pattern do not only occur in the viral, but also in the host cell genome. Hypermethylation of host cell tumor suppressor genes including E-cadherin (CDH1), Cell Adhesion Molecule 1 (CADM1) and Death-Associated Protein Kinase 1 (DAPK1) was reported in recent studies [39–43]. Moreover, emerging evidence suggests that the expression of host cell miRNAs can be affected by altered methylation levels in the respective miRNA promoter region, as well as by the expression of the HPV oncogenes [44]. Both oncoproteins also affect the DNA methylation machinery, as HPV 16 E6 upregulates DNMT1 expression by suppressing p53 and HPV 16 E7 directly binds and activates DNMT1 [45, 46]. Therefore, overexpression of E6 and E7 seems to contribute to the increase in viral and host genome methylation levels. Based on these observations application of demethylating agents would represent an attractive strategy to reverse the effects of hypermethylation potentially repressing HPV oncogene expression and inhibiting neoplastic growth.

The idea of using cytidine analogs like DAC as cytotoxic agents against hematopoietic malignancies was developed about 50 years ago [47]. However, their demethylating potential by incorporation into the DNA and by irreversibly binding to DNMT1 was only discovered almost 15 years later [29–31]. In 2006, the FDA approved DAC (decitabine) for the treatment of MDS, a class of hematopoietic stem cell disorders that is characterized by ineffective hematopoiesis and peripheral blood cytopenia [48]. Despite the strong focus of research studies and clinical trials on hematological malignancies, there are also some trials investigating the effects of DAC in the treatment of solid tumors [32]. In these studies the response of patients to systemically administered DAC varied substantially between different types of cancers.

In the current study, we systematically analyzed the effects of DAC treatment on HPV oncogene expression, the levels of the cell cycle inhibitors p53 and p21 as well as on the neoplastic growth in a panel of HPV-transformed cervical and HNSCC cell lines. DAC treatment reduced proliferation and repressed colony formation in all tested cell lines. Similar to previous studies, the decrease in proliferation after DAC treatment for 72 hours was only moderate in SiHa cells, however, the effect was much more significant after extending the treatment to 120 hours [49, 50]. Several mechanisms seem to be involved in blocking the proliferation under DAC treatment. First, the covalent binding of DNMT1 to DAC-substituted DNA was reported to be cytotoxic independent of DNA demethylation [51]. Second, global DNA demethylation was shown to affect gene expression in the cells, which seems to upregulate tumor suppressor genes and to induce differentiation [30, 52]. Third, DAC treatment resulted in reduced E6 and E7 expression and increased p53 and p21 levels affecting proliferation, as HPV-transformed cells are addicted to the continuous expression of both oncogenes and as p53 and p21 inhibit cell cycle progression. Decreased HPV 16 oncogene expression in CaSki and UM-SCC-47 cells after DAC treatment was also reported in previous studies [38, 50]. In addition, we observed reduced HPV oncogene expression in SiHa, UM-SCC-104, C4-1 and SW756 cells. In SiHa cells the effect of DAC treatment on the HPV 16 oncogene expression was moderate, however, decreasing E7 protein levels could be observed. This was in contrast to previous studies that did not observe obvious changes in E6 and E7 expression in SiHa cells after DAC treatment [49, 50].

In addition, we investigated potential mechanisms explaining the downregulation of E6 and E7 in response to DNA demethylation. Decreased methylation of E2BSs restoring the regulatory role of the viral E2 protein might explain oncogene silencing in lesions containing viral copies with intact E2 open reading frame. In line with this hypothesis and with the report published by Fernandez *et al*., we observed decreased E6 and E7 levels after DAC treatment in CaSki cells that contain tandemly integrated HPV 16 genomes with intact E2 open reading frame and highly methylated HPV 16 URR [38]. However, HPV oncogene expression, proliferation and colony formation were similarly reduced in other HPV-transformed cell lines, which either show low E2BS methylation levels or disrupted E2 open reading frames [53, 54]. These observations point to additional mechanisms that may be involved in the repression of HPV oncogene expression under DAC treatment. Li et al. recently reported that suppression of the viral oncogenes E6 and E7 by themselves interferes with the expression and function of DNA methyltransferases, suggesting that the oncogenic function of the viral oncogenes might in part also be mediated by epigenetic regulation of important tumor suppressor genes [55]. Treatment of HPV-transformed cells with DAC may hence also reverse these direct epigenetic activities of the viral oncogenes independent of the effects that DAC is mediating on their expression.

Our data suggest that the reactivated expression of miR-375 under DAC treatment might be involved in targeting and subsequently degrading E6 and E7 transcripts in HPV-transformed cell lines. Expression of miR-375 was shown to be silenced by promoter hypermethylation which is frequently observed in HPV-transformed malignancies [27]. The tumor suppressive role of miR-375 seems to be not only mediated by its ability to interact with E6 and E7 transcripts, but also by the targeting of host cell mRNAs preventing the expression e.g. of the transcription factor SP1 that was reported to contribute to cancer development and progression [25, 56, 57], of the ubiquitin-protein ligase E6AP which is involved in the E6-mediated degradation of p53 [24], as well as of the protein CIP2A which was shown to prevent the proteolytic degradation of MYC, a transcriptional repressor of p21 [58, 59]. Furthermore, miR-375 also causes the transcriptional repression and prevents the nuclear translocation of telomerase reverse transcriptase (TERT) [24]. By downregulating these factors, miR-375 might also affect proliferation and colony formation independent of degrading the HPV E6 and E7 transcripts. However, the reduction of E6 and E7 levels and the decrease in proliferation could not be directly correlated with the reactivation of miR-375 expression under DAC treatment suggesting that a combination of different mechanisms activated by DAC seem to be involved in mediating these effects.

Overall, the data presented here suggest that treatment of HPV-transformed cells with DAC seems to be a potent approach to repress proliferation and colony formation. To transfer this approach to the patients a systematic evaluation in clinical trials as well as the further study of affected pathways is needed. This might include the analysis of immunological pathways, as DAC treatment was reported to affect endogenous tumor antigen expression and presentation [60–62]. Taken together, the application of demethylating agents might represent a promising approach to treat and possibly even prevent neoplastic diseases triggered by persistent HPV infections.

## MATERIALS AND METHODS

### Human cancer cell lines and 5-aza-2′-deoxycytidine treatment

CaSki cells [63] were acquired from CLS Cell Lines Service GmbH. Authentication of the other cervical cancer cell lines SiHa [64], C4-1 [65] and SW756 [66] were confirmed by Multiplex human Cell line Authentication Tests (Multiplexion). All cervical cancer cell lines were cultured in DMEM (Gibco) containing 10% FBS (Gibco) and 1% Penicillin/Streptomycin (Gibco). The HNSCC cell lines UM-SCC-47 [67] and UM-SCC-104 [68] were developed and kindly provided by Professor Thomas E. Carey (University of Michigan) and cultured in RPMI (Gibco) supplemented with 10% FBS (Gibco), 1% MEM NEAA (Gibco), 1% Glutamine (Gibco) and 25 μg/mL Gentamicin (Gibco).

Depending on the doubling time and the cell size 5 × 10^5^–5 × 10^6^ cells were seeded and cultured without treatment for 24 hours to adhere. Cells were then either treated with 5.6 mM DMSO (Serva) or 5-aza-2′-deoxycytidine (Sigma) dissolved in DMSO at different concentrations ranging from 0.1 – 1.0 μM. Every 24 hours half of the medium was changed and fresh DMSO or DAC was added. This procedure was repeated until the cells were treated for a total of 72 hours reaching a maximum confluence of 70–80%.

### Crystal violet staining and proliferation assay

For stainings with crystal violet the cells were treated with DAC for 72 hours as described in the previous section. Afterwards, the cells were seeded in a 6-well plate format and cultured without further treatment for an additional week. Next, the cells were washed with PBS (Gibco) and stained with crystal violet staining solution consisting of H_2_0, 25% Ethanol (Sigma Aldrich), 1% Formaldehyde (Sigma Aldrich), 0.125% NaCl (Sigma Aldrich), 0.25% Crystal Violet (Sigma Aldrich) for 60 seconds. Crystal violet stained cells were then washed with PBS (Gibco) and pictures were taken to analyse the number of re-grown colonies.

Cell proliferation was measured using the CyQuant^®^ NF Cell Proliferation Assay Kit (Invitrogen) according to the manufactures protocol. The assay determines proliferation by indirectly measuring the DNA content via fluorescent dye binding. For this, 1000 cells were seeded into 96-well plates, treated according to the treatment protocol described above and proliferation was quantified afterwards.

### Transfection of miR-375 in CaSki and SiHa

Transfection of CaSki and SiHa cells was performed in 6-well plates using Lipofectamine 2000 (Invitrogen) as described in the protocol of the manufacturer. Hsa-miR-375 mimics as well as non-specific negative controls (Mission^®^ miRNA, Negative Control 1) were purchased from Sigma Aldrich. After transfection the cells were incubated at 37°C and 5% CO_2_ for 48 hours.

### E6*I and E7 expression analysis, methylation-specific qPCR and miR-375 detection

RNA extraction from cell lines was performed using the RNeasy Mini Kit (Qiagen) including DNaseI (Invitrogen) treatment according to the manufacturer`s instructions and the concentration was spectrophotometrically assessed by measuring the absorbance at A260/280 (NanoDrop 1000). For reverse transcription of mRNA into cDNA, the SuperScript^®^ II Reverse Transcriptase Kit (Invitrogen) was used as described in the manufacturer`s protocol. Each reaction consisted of 1 μg of total RNA, 4 μl of 5x RT buffer, 2 μl of 0.1 M dithiothreitol (DTT), 0.5 μl of 0.5 μg/μl oligo(dT) primers (Invitrogen), 0.5 μl of 0.5 μg/μl single-stranded random hexanucleotides (Bioron), 1 μl of 10 mM dNTPs (Invitrogen) and 0.5 μl of SuperScript^®^ II Reverse Transcriptase (200 U/μl), and was incubated at 37°C for 15 minutes, at 42°C for 60 minutes and at 90°C for 5 minutes.

Quantitative PCR (qPCR) was performed utilizing the Applied Biosystems StepOne™ Real-Time PCR system using Absolute qPCR SYBR Green ROX Mix (Thermo Scientific). Primers used for PCR amplification are listed in Table 2. Cycling conditions were 95°C for 15 minutes and 40 cycles of 95°C for 15 seconds, 60°C for 30 seconds and 72°C for 30 seconds. Melting curves were included in each run to check for amplification specificity. Actin mRNA levels were quantified as a reference control and all samples were run in triplicates. Relative mRNA expression levels were determined by performing the ΔΔCt method and the fold change was calculated as 2^−ΔΔCt^.

**Table 2:** Primer sequences used for methylation-specific and RT qPCR as well as for pyrosequencing

Name	Forward (5′ - 3′)	Reverse (5′ - 3′)	Amplicon (bp)
Primers for RT-qPCR			
HPV 16 E6*I	ACT GCG ACG TGA GGT GTA TTA AC	TGG AAT CTT TGC TTT TTG TCC	85
HPV 16 E7	CAG CTC AGA GGA GGA GGA TG	GCC CAT TAA CAG GTC TTC CA	166
HPV 18 E6*I	TGT ATA TTG CAA GAC AGT ATT	GCT GGA TTC AAC GGT TTC TGG	249
HPV 18 E7	CCC CAA AAT GAA ATT CCG GT	GTC GCT TAA TTG CTC GTG ACA TA	51
β-actin	ATG TGG CCG AGG ACT TTG ATT	AGT GGG GTG GCT TTT AGG ATG	107
Primers for methylation-specific qPCR			
Hsa-miR-375 gene	GGG GCG TTG TGT AGT ATT GAG TTC	GAA ACG AAA ACG AAA AAC CCG	91
β-actin gene	TGG TGA TGG AGG AGG TTT AGT AAG T	AAC CAA TAA AAC CTA CTC CTC CCT TAA	133
Primers for bisulfite-based pyrosequencing of HPV 16 E2BS 3 and 4			
Amplification primers	TTG TAA AAT TGT ATA TGG GTG TG	Bio- AAA TCC TAA AAC ATT ACA ATT CTC	180
Sequencing primer	AAT TTA TGT ATA AAA TTA AGG G		

Hsa-miR-375 promoter methylation was analysed using methylation-specific qPCR. For this, genomic DNA was bisulfite converted as described in the following sections and the modified as well as methylated miR-375 promoter sequence was amplified in subsequent qPCR. Bisulfite converted and unmethylated sequences of β-actin were amplified as a reference control for DNA quality and efficient DNA modification. Primer sequences are listed in Table 2 and were previously published in [27].

For the analysis of hsa-miR-375 expression TaqMan qRT-PCR was performed using TaqMan^®^ MicroRNA Assays (Applied Biosystems) as described in the manufacturer`s instructions. Briefly, the TaqMan^®^ MicroRNA Reverse Transcription Kit (Applied Biosystems) was used for reverse transcription of 50 ng total RNA together with hsa-miR-375 and snRNA U6 specific stem-loop RT primers according to the TaqMan^®^ MicroRNA Assays (Applied Biosystems) protocol. Reactions were incubated at 16°C for 30 minutes, at 42°C for 30 minutes and at 85°C for 5 minutes.

Subsequently, TaqMan^®^ Universal PCR Master Mix (Applied Biosystems) was utilized for qPCR which was run using the following cycling conditions: 95°C for 10 minutes and 40 cycles of 95°C for 15 seconds and 60°C for 60 seconds. All samples were run in triplicates. Expression of snRNA U6 was measured to normalize hsa-miR-375 levels in each sample. Relative microRNA expression levels were determined by performing the ΔΔCt method and the fold change was calculated as 2^−ΔΔCt^.

### Western blot analysis

For lysate preparation cell pellets were dissolved in RIPA buffer (Sigma Aldrich) supplemented with protease inhibitor (Sigma Aldrich), sonicated for 10 seconds, incubated for 30 minutes on ice and then centrifuged at 13,200 rpm for 15 minutes at 4°C. The supernatant was collected and the protein concentration was quantified by performing Bradford Assay using Quick Start ™ Bradford 1x Dye Reagent (BioRad).

Proteins were separated on a 4–20% polyacrylamide gel (BioRad) and blotted onto a PVDF membrane. Subsequent steps were conducted using the Novex Western Breeze Chemiluminescent Immunodetection System (Life technologies) according to the instructions of the manufacturer. Briefly, the PVDF membrane was blocked for 1 hour at room temperature followed by incubation with the primary antibody over night at 4°C. All primary antibodies used in this study were diluted as described in the datasheets of the manufacturers: mouse anti-HPV 16 E7 (NM2, Santa Cruz Biotechnology), mouse anti-p53 (DO-1, Santa Cruz Biotechnology), rabbit anti-p21 (C-19, Santa Cruz Biotechnology)and mouse anti-actin (Clone 4, MP)

Incubation with the secondary antibody coupled to alkaline phosphatase was performed for 1 hour at room temperature. After adding Novex AP Chemiluminescent Substrate CDP Star^®^ (Life technologies) onto the membrane light sensitive films (Lumi-Film Chemiluminescent Detection Film, Roche Diagnostics) were used to detect PVDF membrane bound secondary antibodies. Protein expression levels were normalized to actin.

### DNA isolation, Bisulfite treatment and Pyrosequencing

DNA was isolated using Qiagen's Blood & Cell Culture DNA Mini Kit according to the manufacturer`s instructions. Subsequently, 200–500 ng DNA was bisulfite treated utilizing the Methylamp DNA Methylation Kit (Epigentek) according to the manufacturer`s protocol.

Methylation of 4 CpGs in the HPV 16 URR (reference HPV 16 sequence AF125673.1), encompassing CpG sites within the E2BS 3 and 4 (Figure 3A), was quantitatively analysed by bisulfite pyrosequencing as described previously [69]. The primer pair covering the proximal E2BSs 3 and 4 was used for amplification. Global CpG methylation analysis was performed using PyroMark LINE-1 reagents (Qiagen). Pyrosequencing was carried out using the PyroMarkTM Q24 instrument (Qiagen) according to the manufacturer`s protocol. Assay setup, sequence run and analysis were performed utilizing the PyroMarkTM Q24 Software.

## SUPPLEMENTARY FIGURE AND TABLES


