# Supplementary material for: 5-aza-2′-deoxycytidine (DAC) treatment downregulates the HPV E6 and E7 oncogene expression and blocks neoplastic growth of HPV-associated cancer cells
Source: Oncotarget. 2016 Jul 16;8(32):52104–17. doi: 10.18632/oncotarget.10631 (PMC5581016; doi:10.18632/oncotarget.10631)
Supplement: Supplementary file 1 [file oncotarget-08-52104-s001.pdf]

# 5-aza-2′-deoxycytidine (DAC) treatment downregulates the HPV E6 and E7 oncogene expression and blocks neoplastic growth of HPV-associated cancer cells

## Supplementary Materials

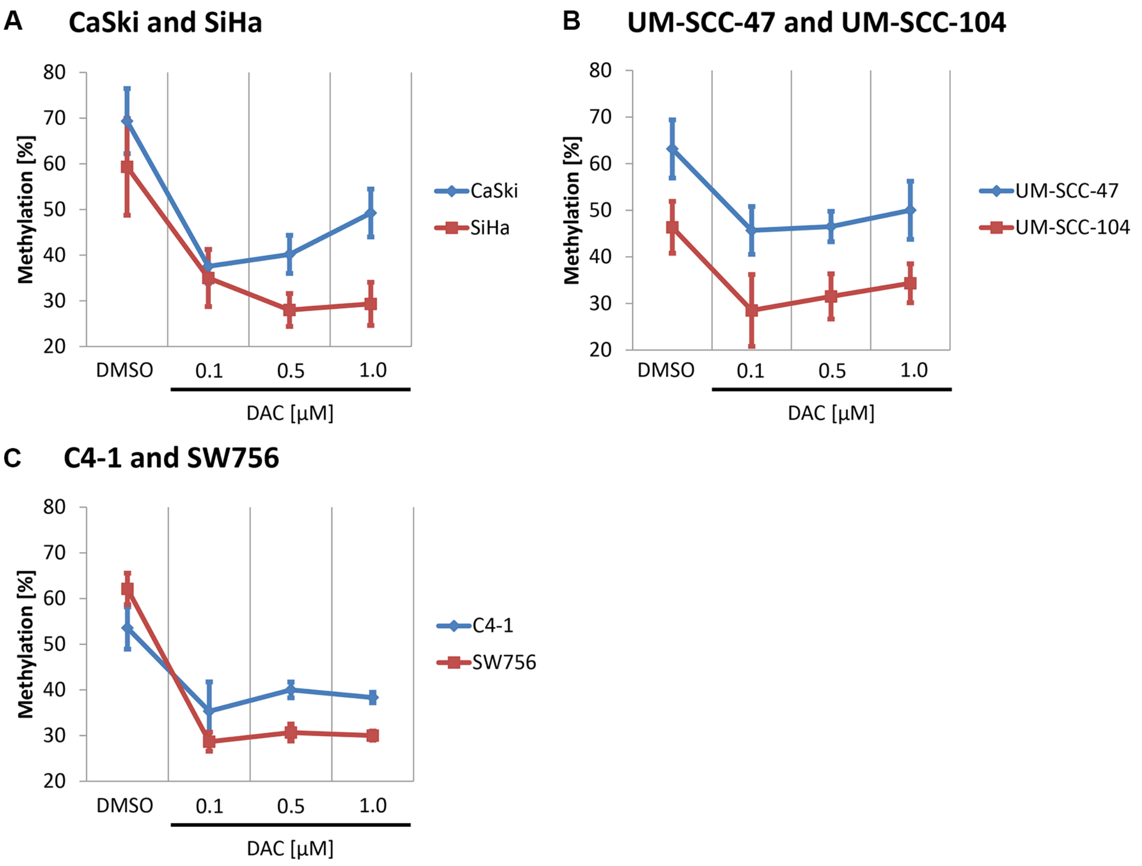


**Supplementary Figure S1: Decrease of LINE-1 methylation levels after DAC treatment indicates global demethylation.** The diagrams show the mean methylation percentage of three CpG dinucleotides located in the LINE-1 transposable element after treating (**A**) CaSki and SiHa, (**B**) UM-SCC-47 and UM-SCC-104 as well as (**C**) C4-1 and SW756 with different concentrations of DAC ranging from 0.1 µM to 1.0 µM. LINE-1 methylation levels were determined by bisulfite treatment of isolated DNA and subsequent pyrosequencing using PyroMark LINE-1 reagents (Qiagen).

**Supplementary Table S1: Densiometric quantification of Western blot bands presented in Figure 1B**

| Fold change | CaSki | | | UM-SCC-47 | | | C4-1 | | |
|---|---|---|---|---|---|---|---|---|---|
| | E7 | p53 | p21 | E7 | p53 | p21 | E7 | p53 | p21 |
| DMSO | 1 | 1 | 1 | 1 | 1 | 1 | 1 | 1 | 1 |
| 0.1 µM DAC | 0.85 | 11.34 | 2.54 | 0.09 | 7.38 | 2.95 | 0.59 | 80.05 | 3.17 |
| 0.5 µM DAC | 0.24 | 14.74 | 3.33 | 0.16 | 6.28 | 3.13 | 0.60 | 138.60 | 3.68 |
| 1.0 µM DAC | 0.05 | 17.07 | 3.39 | 0.05 | 2.53 | 0.89 | 0.57 | 111.45 | 2.95 |

| Fold change | SiHa | | | UM-SCC-104 | | | SW756 | | |
|---|---|---|---|---|---|---|---|---|---|
| | E7 | p53 | p21 | E7 | p53 | p21 | E7 | p53 | p21 |
| DMSO | 1 | 1 | 1 | 1 | 1 | 1 | 1 | 1 | 1 |
| 0.1 µM DAC | 0.68 | 5.20 | 7.75 | 0.27 | 43.71 | 1.34 | 0.93 | 2.63 | 1.70 |
| 0.5 µM DAC | 0.32 | 13.26 | 8.15 | 0.06 | 63.93 | 2.65 | 0.25 | 4.98 | 1.84 |
| 1.0 µM DAC | 0.28 | 12.86 | 9.66 | 0.08 | 112.97 | 4.59 | 0.07 | 6.18 | 2.11 |

**Supplementary Table S2: Densiometric quantification of Western blot bands presented in Figure 6**

| Fold change | HPV 16 E7 | |
|---|---|---|
| | CaSki | SiHa |
| Untreated | 1.29 | 1.38 |
| Non specific control | 1 | 1 |
| miR-375 mimics | 0.35 | 0.07 |
